# Adaptive control of DC-DC power converter: design and experimental investigation with constant power load

**DOI:** 10.1038/s41598-025-29009-y

**Published:** 2025-12-01

**Authors:** Tousif Khan Nizami, Sasank Das Gangula, Ramanjaneya Reddy Udumula, Arghya Chakravarty, Mrutyunjaya Mangaraj, Fareed Ahmad, Arigela Satya Veerendra

**Affiliations:** 1 Department of Electrical and Electronics Engineering, School of Engineering and Sciences, SRM University-AP, Amaravati, 522 240 Andhra Pradesh India; 2 Department of Electrical Engineering, SND College of Engineering and Research Center, Nasik, India; 3 Department of Electrical and Electronics Engineering, Manipal Institute of Technology, Manipal Academy of Higher Education, Manipal, 576104 Karnataka India

**Keywords:** Constant power loads (CPL), Negative impedance characteristics, Adaptive control, Buck converter, Grid stability, Energy science and technology, Engineering

## Abstract

Constant power load (CPL) is a representation of dynamic loads such as power converters and electric motor drives to the DC-DC converter. Precise voltage regulation is necessary for such a combination of loads in a DC microgrid setup. In contrast to typical resistive loads, these loads exhibit negative impedance characteristics, which can cause instability in the DC microgrid system. The proposed adaptive backstepping control methodology effectively solves stability problems while yielding a smooth and fast response of the output voltage and inductor current to changes in resistive load and CPL combination. The success of the proposed method is confirmed by extensive real-time experiments carried out on the prototype of 120*W* converter using the dSpace DS1104 platform. Additionally, to demonstrate the strength of the proposed technique, a thorough comparison is made with the systematically designed cascade-proportional-integral (PI) and the sliding mode based control systems, which clearly indicate the proposed control is found to be $46\%$ and $60\%$ faster compared to cascade PI and sliding mode control methods respectively. Thus showcasing the potential of proposed control technique for real-time CPL applications.

## Introduction

**Fig. 1:**
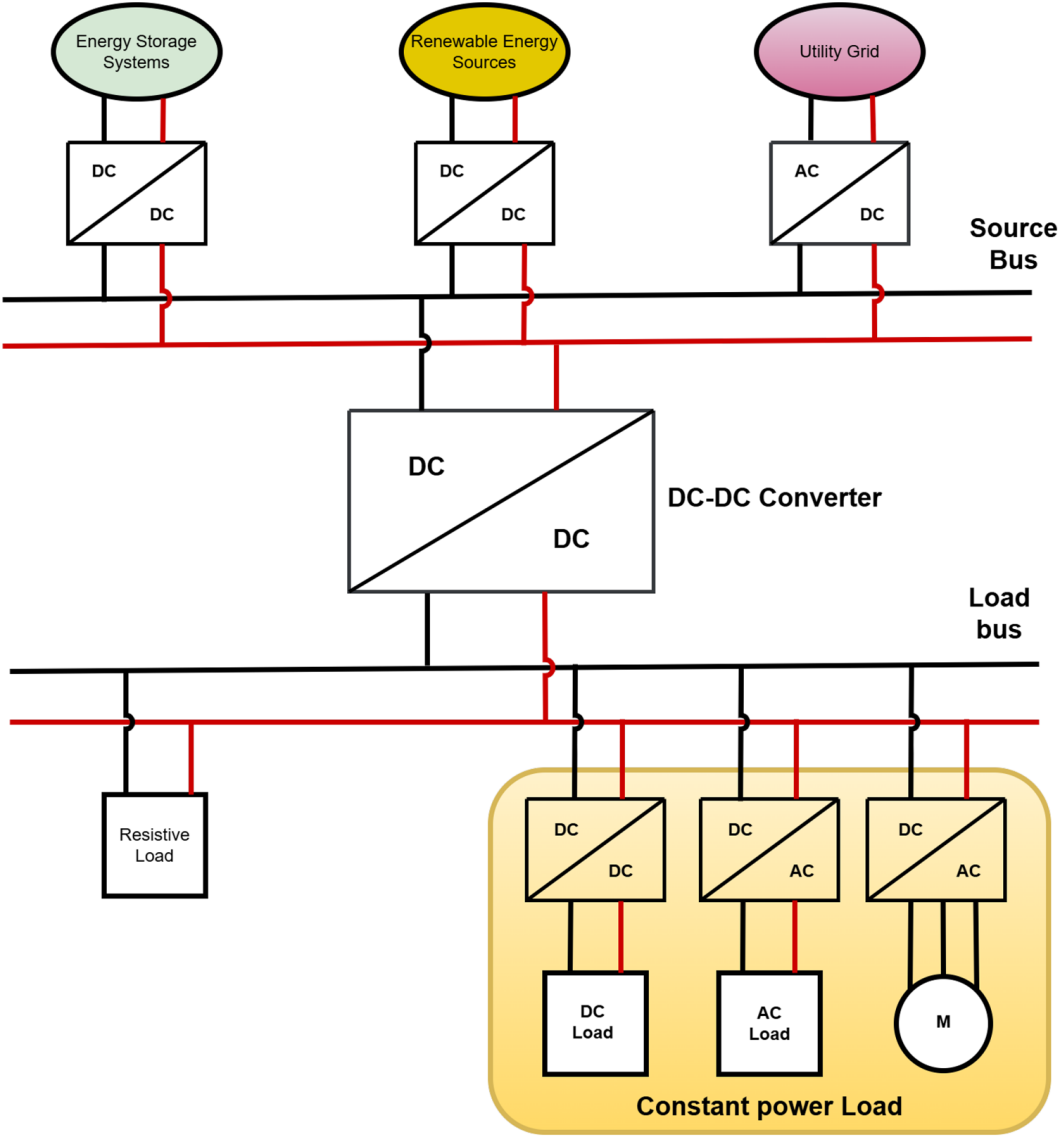
Illustrative block diagram of a DC Micro grid set-up.

DC–DC power electronic converters are increasingly being utilized in a variety of applications. These include the international space station, spacecraft, telecommunications, automotive power grid, robotics, ships and medical electronics etc. These power converters are also employed in particularly DC micro grid applications, where renewable energy sources (RES) are connected to the DC source bus^1–5^. Figure 1 shows the fundamental block diagram of a DC microgrid (DCMG) configuration, where RES and units for energy storage are connected to the utility grid through the DC-link source bus. At the load bus, the CPLs and resistive loads are connected to the DC-link. The interconnections between various energy sources and the load, can lead to several dynamic interactions^6^. Such interconnections between these two DC-links requires the use of DC-DC power conversion with suitable control systems to ensure a fast, smooth, and efficient power flow.

DC microgrids provide distinct benefits over AC systems. They omit the problems like reactive power losses and enhances the power conversion efficiency. In addition, DCMGs do not require frequency synchronization^7^. This boosts the overall resilience of the system. Since, DCMGs permit, direct connection of loads at different voltage levels by using multiple DC-DC converters, hence their independent control is a must. Moreover, the fact that various sources, such as solar panels and batteries, do not exhibit ideal voltage source characteristics. As a result, the design of an efficient and robust control systems for DC-DC converters is a crucial task, to avert source and load disturbances in DCMGs. Other examples of energy distribution through multi-power converter setups are electric vehicles, satellites, and underwater vehicles which require an adequate control system^8–11^.

**Fig. 2:**
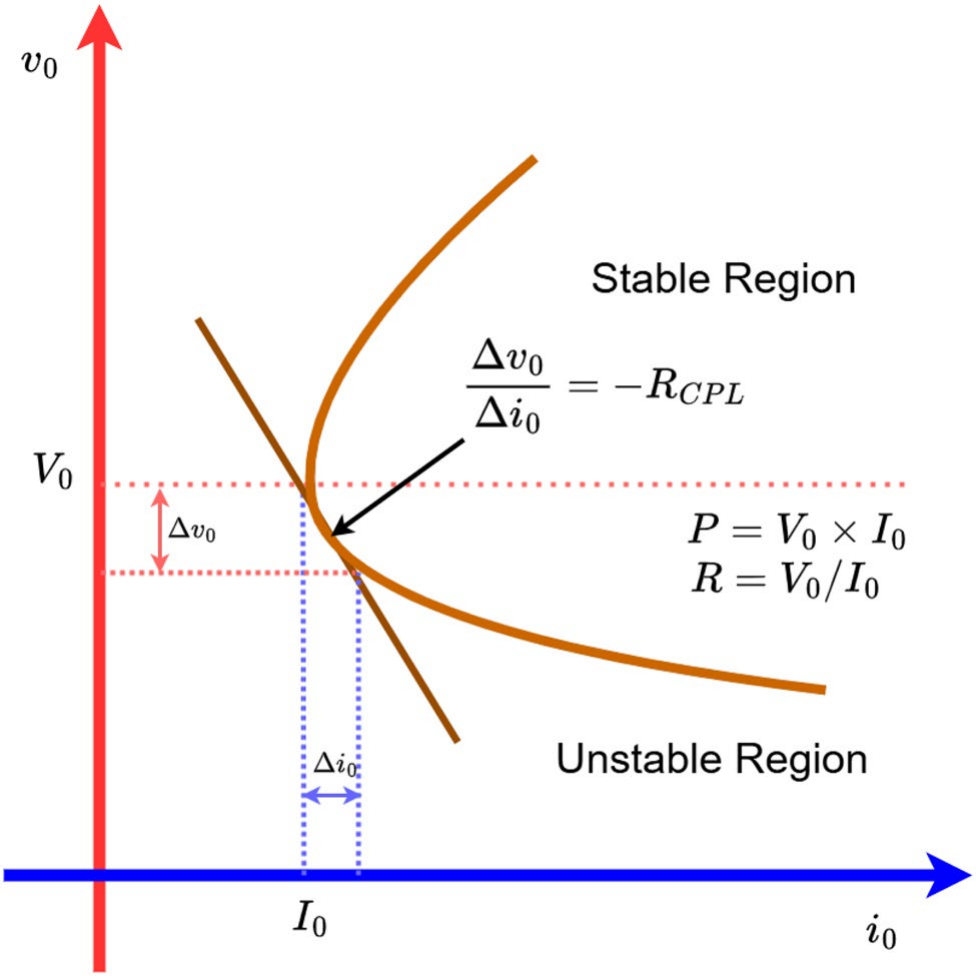
The $v-i$ characteristics of the equivalent load for a DC/DC converter that is powering a resistive load and a CPL^17^.

Typically, load types are categorized into constant resistive loads and CPLs^12^. These classifications are based on the characteristics of how the load behave in response to changes in voltage or power demands. DCMGs operating with CPLs may experience stability challenges, particularly due to their multi-converter configuration^13–16^. As indicated in Fig. 2, the voltage across the terminals of CPL decreases, it draws high current and vice-versa, resulting in a negative impedance within the DCMG set-up, thus significantly impacting on the system’s stability^17,18^. Furthermore, a CPL demands a steady amount of power irrespective of the voltage levels. Its voltage-current ($v-i$) characteristics is nonlinear in nature. This follows $I = P / V$, with *P* as the fixed power. Such a pattern creates negative incremental impedance ($\frac{dI}{dV}=-P/V^{2}$). In the absence of robust control mechanism, such a load behaviour can lead to destabilizing the whole system.

Such a behavior can cause voltage droop^19^, which is when the voltage at the point of common coupling drops as the load draws more current. This causes sudden and significant output voltage change. Nonlinear loads are commonly encountered in tightly regulated motor loads as well as resistive loads supplied through power electronic converters^20^.

Numerous solutions for addressing CPL instability are discussed in the literature. One of the most widely used methods is passive damping technique which involves addition of passive elements at the input filter to reduce the affect of negative incremental impedance. This approach helps to maintain stability by converting a CPL into a resistive load. However, this methodology has several disadvantages. It can lead to trade-off in the design requirements, thus limiting overall flexibility, decrease in system efficiency, increase size and the cost^21,22^.

Active damping^23^ addresses instability by making adjustments to the control loop to create a damping effect. This can be obtained by affixing an electronic device to inject the compensating current. There are three main methods for implementing active damping^24^. The first approach is source-side active damping method. This method involves using an extra control loop at the source subsystem for compensation. This approach enhances the output impedance of the source converter, ensuring it complies with stability requirements. However, this technique is not suitable for systems with an LC filter at the input stage. Moreover, source-side active damping boosts stability. It does this by changing the output impedance of the source converter. Yet it has clear downsides. First, damping based on linear feedback works well only near the intended operating point. This makes it weak against large variations in CPL power. Second, an LC filter on the source side can lead to issues. Besides, the method works only in limited frequency range and requires extra sensors^13,22^.

To counter instability, active damping at the load-side is suggested when the source of the CPL includes a LC filter. This approach operates by introducing current or power in the closed loops of CPL to adjust with the input impedance, while ensuring that Middlebrook’s stability criteria is satisfied. The third approach involves the addition of an auxiliary device that supplies the necessary compensating current well within the system’s operating range. This virtual impedance^25^, also known as virtual resistance, is a technique for active damping that has been suggested by researchers in the past. This method strengthens the system’s transfer function and helps to move the poles into a more stable area, specifically to the left side of the s-plane. In design and analysis, conventional passive and active damping techniques usually depend on small signal analysis. However, these methods become less effective when faced with large disturbances or significant changes in load.

Therefore, the performance of converters, particularly in the presence of CPLs, can result in system instability due to inherent non-linearities, making closed-loop control critical to overcome this challenge^26^. The authors in Ref. 27 presents a PID controller that employees the Kharitonov theorem to tackle stability challenges in DC power systems with CPLs affected by uncertainties in input voltage, and various load disturbances. The Hermite-Biehler theorem helps identify the stabilization sets for the PID controller, which guarantees stability across different operating conditions. Numerical results indicate that the proposed linear controller achieves near-satisfactory tracking performance. However, the study lacks real-time experimental work showcasing parametric variations in the converter system feeding CPL.

Several nonlinear control methods have been developed to counter the instability issues caused by CPL. Fuzzy logic control (FLC)^28^ can manage converter operations in hybrid energy systems. It has shown to be effective, especially in complex systems. However, creating fuzzy logic rules and membership functions is challenging. The complexity and time needed to fine-tune control parameters represents a significant trade-off compared to traditional control methods. Additionally, FLC largely depends on the control designer’s expertise. This means that while FLC is a versatile and effective tool, its effectiveness can differ based on the approach of the design.

In Ref. 29, a nonlinear control method is proposed for the DC-DC buck power converter that drives the CPL. It combines feedback control with feedforward compensation and uses a nonlinear reduced-order state observer to approximate the power of CPL and its dynamics over time. This method relies on feedback linearization, which assumes that the load power and its change rate are known. This controller functions satisfactorily without requiring a disturbance sensor. Nonetheless, this approach has some limitations, such as reduced accuracy in tracking and high computational burden, thus impacting the processing speeds. Similarly this type of control^30^ method has been applied for DC-DC boost converter with CPL.

In Ref. 31, shows an improved voltage control in DC-DC buck converters driving CPL by using a twin delayed deep deterministic policy gradient technique. The experimental tests are used to validate the claims of reliability under different loads and changing parameters. In-spite of such controllers suffering from complexity, training and tuning in real-time is a challenging task.

In Ref. 32 authors designed an adaptive sliding mode control (ASMC) system for buck converters in DC microgrids to handle CPLs and external disturbances. Simulation results show that this approach leads to lesser settling times and less chattering effect, in contrast to the conventional sliding mode control. Output voltage stability under source voltage and load change conditions are presented. In Ref. 33 authors conducted an investigation on a cascade connection of a boost DC-DC converter delivering constant power to buck DC-DC converter using SMC method. However, the chattering issues, increased electromagnetic interference (EMI) in SMC are the limitations. In Ref. 34, a comprehensive study on the design and validation of an H${\infty }$-based controller having a DC-DC buck power converter in a DC microgrid arrangement, focusing on the challenges posed by CPL has been attempted. Backstepping control, (BSC)^35^ is a nonlinear control method popular for its structured approach to designing controllers, simplicity in comprehension and implementation, capacity to address discrepancies in linear parameters, and effective mitigation of parametric uncertainties. Nonetheless, it demands real-time information about the eventualities affecting it. Some works conducted in this direction includes^36–38^, involving boost and buck-boost converters regulated through various control strategies.

In Ref. 39 authors present a sliding mode controller primarily paired with H-infinity secondary controller for islanded microgrid that feed CPL. It ensures precise power sharing and restores DC bus voltage levels. However, the two-layer design impose challenges and demands fine tuning. In Ref. 40, authors propose an adaptive method that uses a cubature Kalman filter to estimate load power in real time. The ANFIS controller then adjusts currents in energy storage units. In Ref. 41 a Brayton-Moser passivity-based controller is proposed for a two-level interleaved boost converter. It handles nonlinear impedance issues and gains improvement in efficiency. In Ref. 42 authors proposes a passivity-based control with disturbance observer for a DC-DC boost converter serving CPLs. However, the design is more complex for implementation.

Therefore, the design and development of an intelligent adaptive control scheme to control the power converter driving CPLs is a challenging and crucial task. In this direction, this article presents adaptive backstepping control (ABSC) technique to counter the following specific challenges posed by constant power and resistive loads while regulating the output voltage; (i) CPL introducing dynamic characteristics (negative impedance) in the load causing output instability, (ii) eventualities faced due to sudden changes in the resistive load and (iii) large scale fluctuations in the source voltage. The ABSC technique proposed in this work for a DC-DC buck power converter not only estimates real-time dynamic and static loads but also ensures tight output voltage tracking, besides ensuring smooth inductor current profile. Additionally, the proposed controller dynamically adjusts the controller parameters to handle fluctuating CPL conditions, guaranteeing asymptotic stability and robust output performance across a wide range of operating points^37^. Since, the efficiency is crucial when dealing with CPLs, the proposed control minimizes the losses and boosts the converter efficiency. Over and above the proposed method offers a recursive and systematic design procedure that can accurately regulate the system voltage in response to the disturbances and even otherwise. The proposed control law is tailored to fast reflect untoward perturbations, if any and to compensate the same effectively to ensure globally asymptotic stability. Further, the experimental tests conducted in real-time on 120*W* converter under start-up, load change and source voltage disturbances verify the effectiveness of the proposed controller. The claims have been further made by fair comparison with systematically designed cascade PI^43^ and SMC techniques on fairgrounds.

The results clearly highlight that the proposed controller efficiently deals with the system’s nonlinearities during both start-up and steady-state converter operations. Results indicate that proposed control is found to be $46\%$ and $60\%$ faster compared to cascade PI and sliding mode control method, while achieving the said objective.

This paper is detailed as follows. Section "Mathematical modeling" describes the mathematical model of the system, and presents control objectives. Section "Control methodology: adaptive backstepping control", introduces the adaptive backstepping control design for DC-DC buck power converter driving CPL. Classical design of cascade-PI control and sliding mode control are also discussed. Section "Experimental results and discussion" offers extensive tests in real-time. It also includes various case studies. These assess how well the proposed control works. Finally, the conclusions are summarized in Sect. "Conclusion".

## Mathematical modeling

**Fig. 3:**
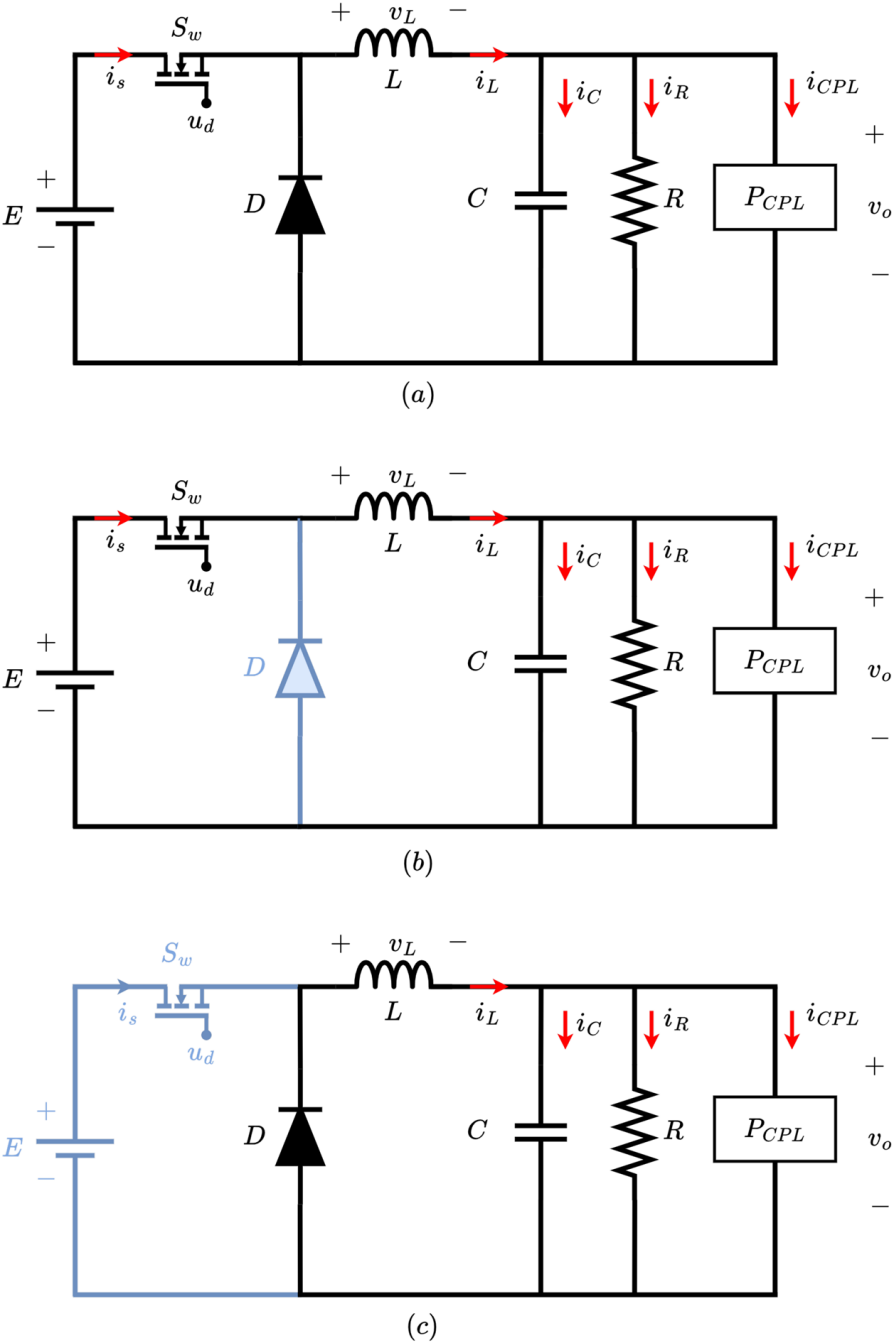
Shows (**a**) DC-DC power buck converter driving CPL and resistive load; (**a**) when the converter is in ON mode and (**b**) when the converter is in OFF mode.

The topology of the non-isolated DC-DC buck converter is presented in Fig. 3a. The converter provides power to a parallel combination of constant resistive load and CPLs. The operation of the DC-DC buck power converter in continuous conduction mode can be characterized by two distinct phases: ON and OFF. These modes are depicted in Figs. 3b and c. The alternating modes of operation are crucial to regulate the output voltage and inductor current requirements of connected CPL and constant resistive loads. In addition, both the converter configuration and switching behavior are significant in determining the overall performance and efficiency in driving the CPL and constant resistive loads.

Herein, *E* represents the DC voltage source that powers the system. Here, the inductor is labeled as *L*, while the capacitor is marked as *C*. The feedback freewheeling diode is shown as *D*. In this arrangement, the constant resistive load is shown as *R*, which usually represents a resistive load. The constant power load is displayed as a CPL block, which is in parallel to the resistive load *R*. The DC-DC buck power converter operates using a MOSFET switch, referred to as $S_{w}$. Here, the current passing via CPL is denoted by $i_{CPL}$. System dynamics are characterized by the ON and OFF states of the converter. In this context, $x_{1}$ indicates the voltage across the capacitor, which is referred to as $v_{0}$. Meanwhile, $x_{2}$ represents the current flowing through the inductor, known as $i_{L}$, with the control input *u* ranging between 0 and 1.

1 $$\begin{aligned} \dot{x}_{1}= & \frac{x_{2}}{C}-\frac{x_{1}}{RC}-\frac{P_{CPL}}{Cx_{1}}, \end{aligned}$$

2 $$\begin{aligned} \dot{x}_{2}= & \frac{Eu}{L}-\frac{x_{1}}{L}, \end{aligned}$$

The objective of this work is to propose a fast learning, efficient and adaptive control scheme for the output voltage regulation, besides guaranteeing smooth transient response of inductor current response in the DC-DC buck converter feeding the dynamically changing constant power and constant resistive loads.

## Control methodology: adaptive backstepping control

**Fig. 4:**
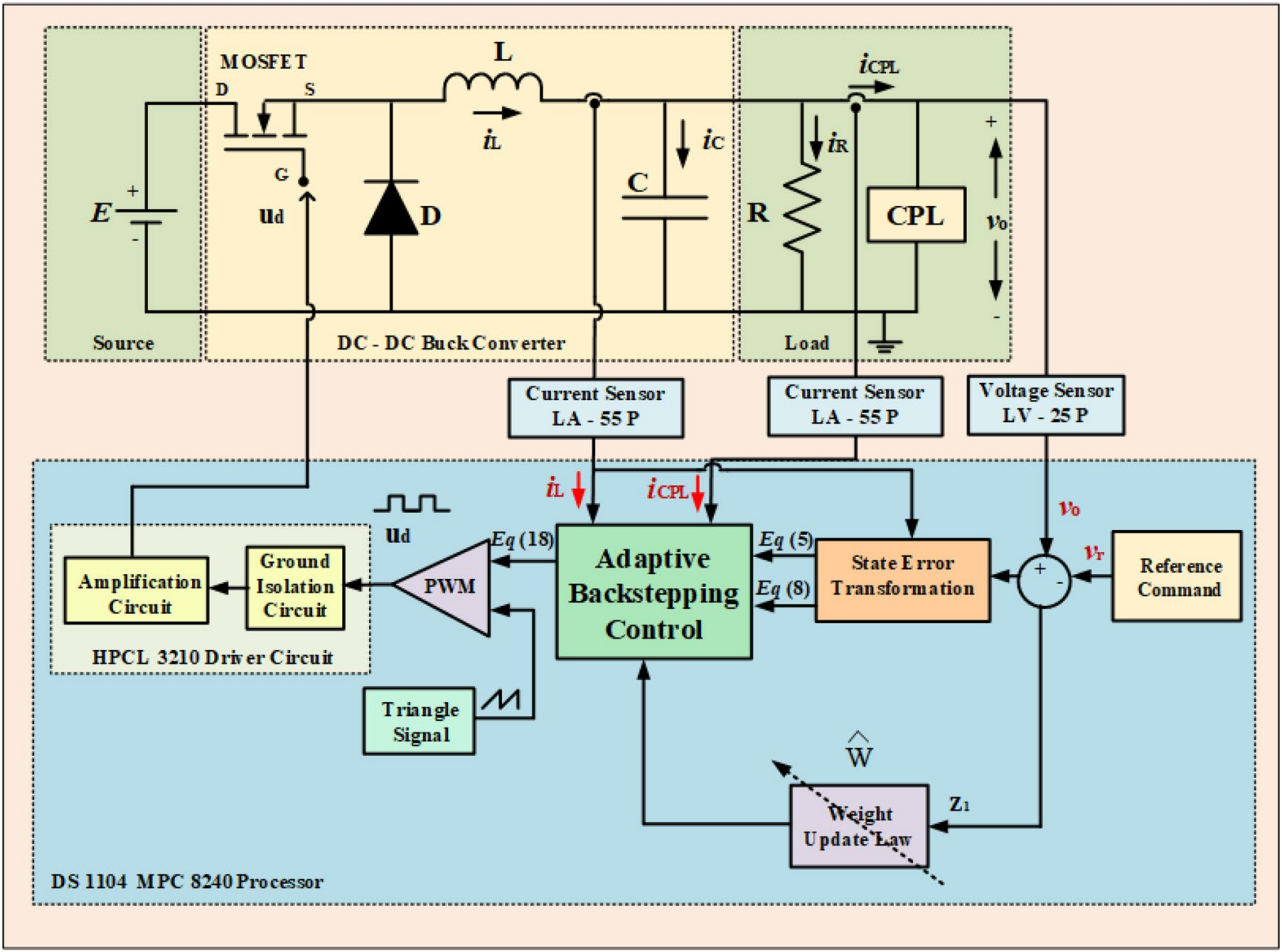
The diagram shows a proposed control system for a DC-DC buck converter that supplies power to both a CPL and a resistive load.

This study presents a control strategy for a DC-DC buck converter that can efficiently power both CPLs and resistive loads. Figure 4 illustrates this proposed control method, highlighting the key components and their interactions.

A buck converter functions by reducing voltage in a DC system while increasing current output. This is crucial for meeting the specific output voltage and current needs of the connected loads. The research here employs an adaptive backstepping control technique to stabilize the converter’s operation and enhance its efficiency for CPLs.

This adaptive backstepping method tackles the challenges of nonlinearities and changing load conditions, which are common in power conversion. By adjusting controller parameters in real-time, the strategy ensures the converter operates reliably and efficiently. Compared to traditional control methods, this approach offers better stability and responsiveness to load changes. Herein, the unknown term $P_{CPL}$ is estimated using the weight vector *W*, as

3 $$\begin{aligned} P_{CPL}/x_{1}=W^{T}\Phi, \end{aligned}$$

where $\Phi$ is the regressor. From Eqs. (1) and (3), we can get;

4 $$\begin{aligned} \dot{x}_{1}=\frac{x_{2}}{C}-\frac{x_{1}}{RC}-\frac{W^{T}\Phi }{C}. \end{aligned}$$

### Controller design

The initial inconsistency variable, $z_{1}$, quantifies the deviation to the actual output voltage, $x_{1}$, and the desired reference voltage, $v_{r}(t)$.

5 $$\begin{aligned} z_{1}=x_{1}-v_{r}. \end{aligned}$$

Differentiating (5) and using (4) yields:

6 $$\begin{aligned} \dot{z}_{1}=\frac{x_{2}}{C}-\frac{x_{1}}{RC}-\frac{W^{T}\Phi }{C}- \dot{v}_{r}, \end{aligned}$$

wherein, the ideal inductor current is denoted with $\alpha$, called as a virtual control input

7 $$\begin{aligned} \alpha =C\left[ \frac{x_{1}}{RC}-\frac{\hat{W}^{T}\Phi }{C}- \dot{v}_{r}-k_{1}z_{1} \right]. \end{aligned}$$

Let us represent the second inconsistency variable $z_{2}$ as the difference between $x_{2}$ and $\alpha$

8 $$\begin{aligned} z_{2}=x_{2}-\alpha. \end{aligned}$$

From (8) and (6) we get,

9 $$\begin{aligned} \dot{z}_{1}=\frac{z_{2}+\alpha }{C}-\frac{x_{1}}{RC}-\frac{W^{T}\Phi }{C}- \dot{v}_{r}. \end{aligned}$$

Further, (9) can be rewritten as

10 $$\begin{aligned} \dot{z}_{1}=\frac{z_{2}}{C}+\frac{\alpha }{C}-\frac{x_{1}}{RC}-\frac{W^{T}\Phi }{C}- \dot{v}_{r}. \end{aligned}$$

By incorporating the virtual control input from Eq. (7) in the above (10), we get

11 $$\begin{aligned} \dot{z}_{1}=\frac{z_{2}}{C}-k_{1}z_{1}-\frac{\tilde{W}^{T}\phi }{C}, \end{aligned}$$

where $k_{1}$ is a design parameter, $\tilde{W}^{T}$ is the difference between the real-time weight and the estimated weight ($\tilde{W}^{T}=W^{T}-\hat{W}^{T}$).

Let us take the first Lyapunov candidate function as

12 $$\begin{aligned} V_{1}=0.5\times z_{1}^{2}+0.5 \times \frac{\tilde{W}^{T}\tilde{W}}{\gamma }. \end{aligned}$$

The time derivative of (12) as

13 $$\begin{aligned} \dot{V}_{1}=z_{1}\dot{z}_{1}+\frac{\tilde{W}^{T}\dot{\hat{W}}}{\gamma }. \end{aligned}$$

From (11) and (13) we get,

14 $$\begin{aligned} \dot{V}_{1}=\frac{z_{1}z_{2}}{C}-k_{1}z_{1}^{2}. \end{aligned}$$

Let us take the second Lyapunov candidate function as

15 $$\begin{aligned} V_{2}=0.5\times z_{2}^{2} \end{aligned}$$

From (2), (8) and (15) we get,

16 $$\begin{aligned} \dot{V}_{2}=\frac{uEz_{2}}{L}-\frac{{x_{1}}{z_{2}}}{L}-\dot{\alpha } z_{2}. \end{aligned}$$

The resulting control law formulated as

17 $$\begin{aligned} u=\frac{L}{Ez_{2}}\Big (-k_{2}z_{2}^{2}+\frac{x_{1}z_{2}}{L}+\dot{\alpha }z_{2}-\frac{z_{1}z_{2}}{C}\Big ), \end{aligned}$$

which further simplifies as follows

18 $$\begin{aligned} u=\frac{L}{E}\Big (-k_{2}z_{2}+\frac{x_{1}}{L}+\dot{\alpha }-\frac{z_{1}}{C}\Big ). \end{aligned}$$

We obtain $\dot{V}_{2}$ as,

19 $$\begin{aligned} \dot{V}_{2}=-k_{2}z_{2}^{2}-\frac{z_{1}z_{2}}{C}. \end{aligned}$$

The total Lyapunov function defined as

20 $$\begin{aligned} V=V_{1}+V_{2}. \end{aligned}$$

Finding time derivative of Eq. (20), we get

21 $$\begin{aligned} \dot{V}=-k_{1}z_{1}^{2}-k_{2}z_{2}^{2}. \end{aligned}$$

It has been shown that a negative semi-definiteness $\dot{V}$ leads to boundedness of the switching control signal. Establishing the asymptotic stability of the tracking error $z_{1}, z_{2}$ necessitates $\dot{V}=0$ and $V=0$ if and only if $z_{1}=0, z_{2}=0$ as $t\rightarrow \infty$. Consequently, it can be deduced that $\ddot{V}$ remains limited. Thus, according to Barbalat’s lemma, $\dot{V} = 0$ as $t \rightarrow \infty$, confirming the stability in asymptotic sense, as $\Vert z\Vert =0$, where $z=[z_{1}, z_{2}]$.

### Cascade-PI control

**Fig. 5:**
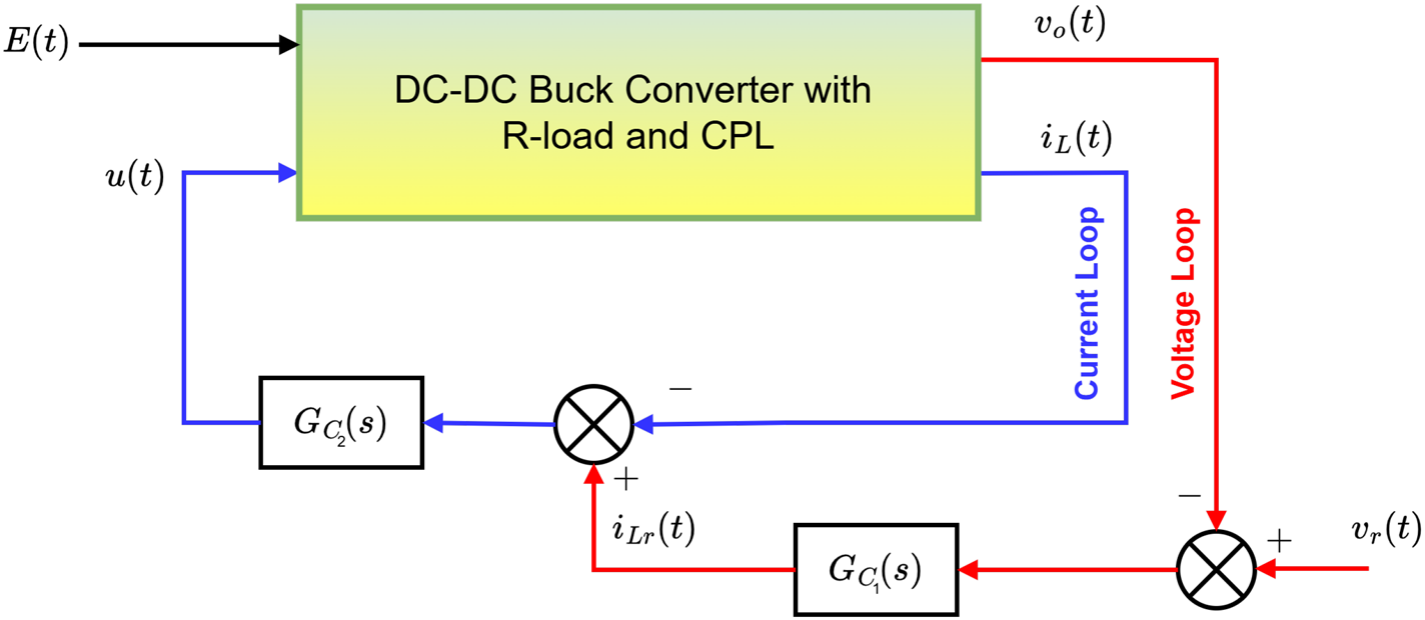
Figure represents a Cascade control system for a DC-DC buck converter.

This sub-section, gives the design of the conventional cascade PI control method based on mathematical modeling, intended for the objective of voltage regulation for buck power converting catering both CPL and constant resistive load has been summarized.

Thereafter, a detailed performance comparisons is conducted to evaluate the merits of the proposed control method. This cascade-PI method, as described in Ref. 43, consists of dual feedback loops. The first loop deals with the output voltage, while the second loop focuses on controlling the current in the inductor. Figure 5 depicts the control block diagram of the cascade-PI control method. Here $v_r(t)$ is the reference output voltage, $i_{Lr}(t)$ is the reference inductor current and *d*(*t*) is the duty for the buck converter.

*Outer voltage loop* Considering the linear Proportional-integral (PI) controller for controlling the output voltage,

22 $$\begin{aligned} G_{C_{1}}(s)= & \frac{K_{1}(RCs+1)}{Rs}, \end{aligned}$$

23 $$\begin{aligned} k_{1}= & \frac{1}{RC}. \end{aligned}$$

Here, undamped natural frequency of the voltage control loop is represented by $\omega _n=\frac{1}{RC}$ is the .

*Inner current loop* Next, considering the current control through PI controller, we get

24 $$\begin{aligned} G_{C_{2}}(s)=\frac{K_{2}(Ts+1)}{s}. \end{aligned}$$

Then the output inductor current under the action of $G_{C_{2}}(s)$ is given by

$$\begin{aligned} I_{L}(s)= & -\frac{s}{Ls^2+K_2Tv_is+K_2v_i}v_o(s) +\frac{K_2(Ts+1)v_i}{Ls^2+K_2Tv_is+K_2v_i}i_r(s). \end{aligned}$$

The current loop takes 20 times faster than the natural frequency voltage loop for the measurement.

25 $$\begin{aligned} \omega _{1}= & N\omega _{n}=\sqrt{\frac{K_2v_i}{L}}; N=20, \end{aligned}$$

26 $$\begin{aligned} k_{2}= & \frac{N^2\omega ^2_nL}{v_i}=484.57, \end{aligned}$$

27 $$\begin{aligned} 2N\omega _n= & \frac{K_2Tv_i}{L}\ \Rightarrow T=\frac{2}{N\omega _n}=7.19\times 10^{-4}. \end{aligned}$$

Hence utilizing (22) to (27) the voltage loop PI controller is found as

28 $$\begin{aligned} G_{C_{1}}(s)=0.013+\frac{1.929}{s}. \end{aligned}$$

Similarly, the inner current loop controller is designed as

29 $$\begin{aligned} G_{C_{2}}(s)=0.348+\frac{484.57}{s}. \end{aligned}$$

Experiments are carried out to confirm how well the cascade controller performs in a closed loop system. This will allow for a meaningful comparison.

### Sliding mode control

Furthermore, this sub-section presents the design and comparison of the proposed control method with one of the nonlinear control techniques based on variable structure systems, the sliding mode control.

Here, the sliding surface has been framed based on the output voltage error and its time derivative^44^, to capture the dynamical changes in the output voltage of the power converter.

The voltage error is taken as $x_{1}$ as

30 $$\begin{aligned} x_{1}=v_{o}-v_{ref} \end{aligned}$$

and then its derivative,

31 $$\begin{aligned} x_{2}=\dot{x_{1}} \end{aligned}$$

the sliding surface is given by,

32 $$\begin{aligned} S=\lambda x_{1}+x_{2} \end{aligned}$$

and the control law for SMC technique is given by

33 $$\begin{aligned} u_{SMC}=0.5\times (1-sign(S)). \end{aligned}$$

## Experimental results & discussion

**Fig. 6:**
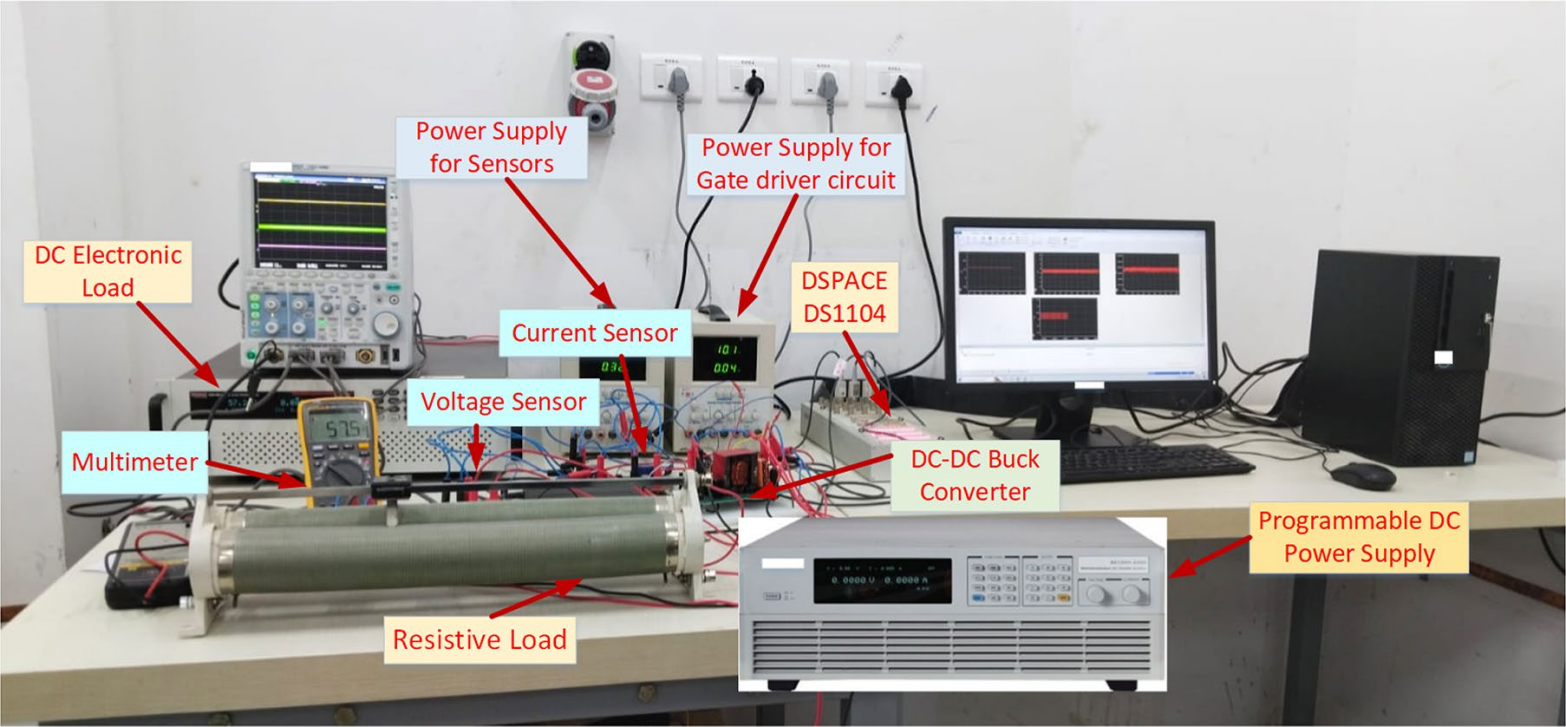
Experimental test bench.

In order to validate the efficacy of the proposed method, an extensive real-time investigations are conducted on a laboratory prototype of 120 W DC-DC buck power converter feeding combination of both constant resistive and CPLs. The same is detailed in this session. Figure 6 showcases the setup of proposed control implementation for the power converter. The control mechanism is implemented on a dSPACE DS-1104 consisting of MPC8240 DSP processor. A programmable DC power supply used for sourcing the input power. It can provide 600V, 8.5A and 5KW power.

The IRF740 power MOSFET is employed for switching requirement. It has a voltage rating of 500 V, a current carrying capacity of maximum 20 A, its resistance when on is $0.27\Omega$. The DC electronic load used, which has been suitably programmed to impact changing power levels of the CPL. The signal sensing is done by integrating Hall effect-based sensors LV-25P with $40\mu s$ sampling rate and LA-55P with $12.5\mu s$ sampling rate, to read the real-time output voltage and inductor current signals. In furtherance, the dSPACE Control Desk DS1104 is linked with the power converter through the sensors via a desktop computer that runs on an Intel$\circledR$ Core${}^{\textrm{TM}}$ i7 processor of 3.2 GHz. MATLAB tool is utilized to implement the controller’s functionality on DSPACE control board. Further, the DSPACE DS1104 control board is used to compute the control pulses to be sent to the semiconductor switch through gate isolation circuit. In this work, the control algorithms is updated at every $20\mu s$. The other detailed specifications of the DC-DC buck converter are outlined in Table 1.

## Remark 1

The real-time implementation was carried out on a laboratory prototype of a 120 W DC–DC buck converter. The converter is designed for an input voltage of 100 V, an output of 60 V, with an inductor of 6.28 mH, a filter capacitor of 100 $\mu F$, a load resistance of $72 \Omega$, and a PWM switching frequency of 10kHz. The switching device employed is an IRF740 MOSFET rated at 500 V and 20 A, with an on-state resistance of $0.27 \Omega$. For sensing, we used LV-25P Hall-effect voltage sensors with a $40 \mu s$ sampling rate and LA-55P current sensors with a $1 \mu s$ sampling rate to measure output voltage and inductor current in real time. A DC electronic load was programmed to emulate dynamic CPL conditions. The control algorithm was executed on a dSPACE DS1104 board, which includes an MPC8240 DSP processor running at 3.2 GHz. The controller was coded in MATLAB/Simulink and updated at $20 \mu s$ intervals.

**Table 1:** Specifications of the DC-DC buck converter.

Parameter	Rating
Input voltage, *E*	100 V
output voltage, $v_{o}$	60 V
Power $P_{max}$	120 W
Inductor, *L*	6.28 mH
Resistior, *R*	$72 \Omega$
Filter Capacitor, *C*	$100\mu F$
PWM Switching frequency, *f*	10 kHz

The adaptive control design constants of the proposed control are set as $k_{1}=60$, $k_{2}=500000$ and the learning parameter as $\gamma =0.001$. The experiment follows specific testing cases, as outlined below.

### Start-up test

**Fig. 7:**
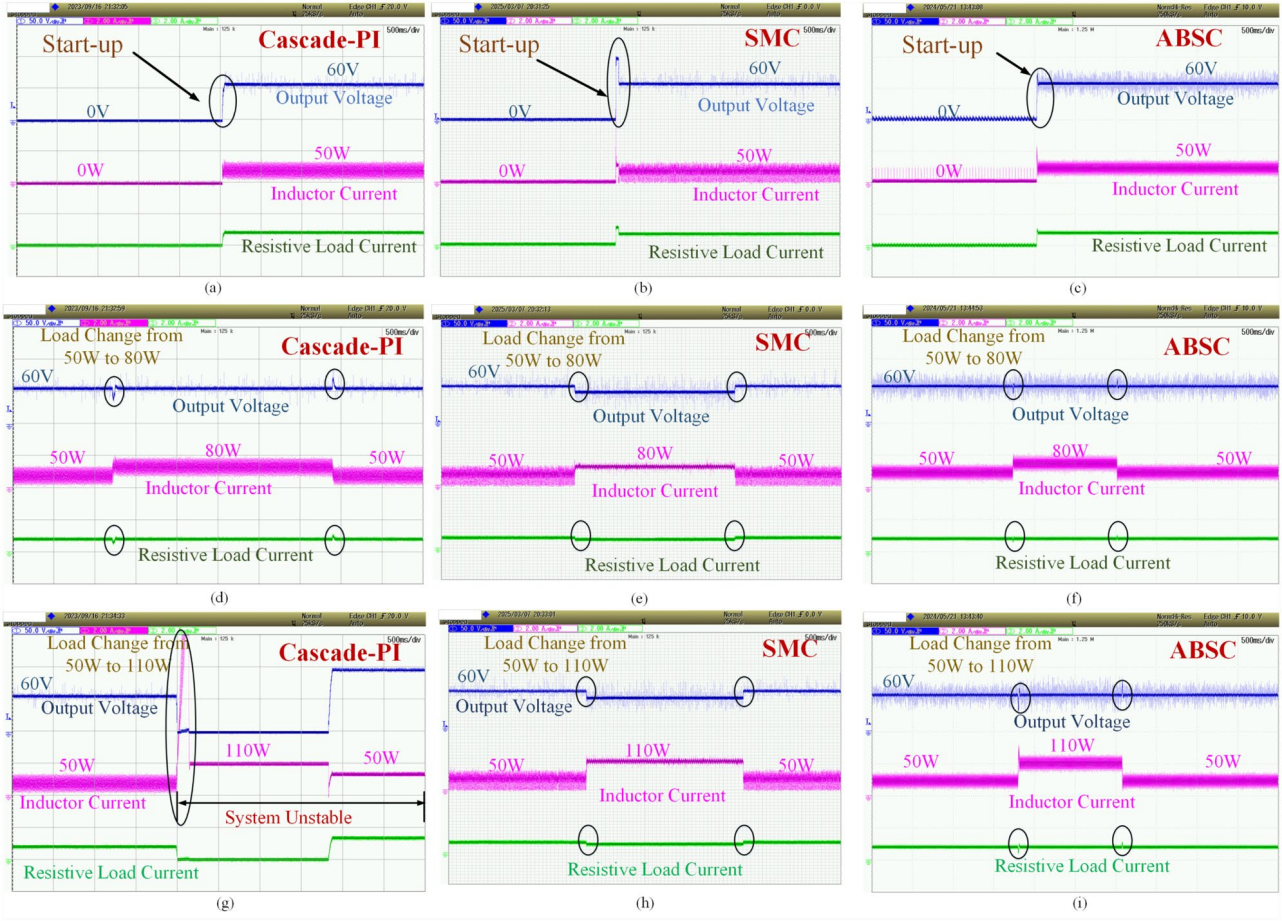
The initial response is for (**a**) cascade-PI, (**b**) SMC and (**c**) proposed ABSC; load shift from 50 to 80 W for the (**d**) cascade-PI, (**e**) SMC and (**f**) proposed ABSC; Lastly, the load shift from from 50 to 110 W is also examined for the (**g**) cascade-PI, (**h**) SMC and (**i**) proposed ABSC.

In this examination, we evaluate the outcomes proposed control, designed SMC and designed cascade PI controller on the closed loop controlled DC-DC buck power converter with constant resitive load and CPL. This research focuses on the initial behaviour of a DC-DC buck converter. We look at its output voltage and the current flowing through the inductor. In this assessment, as shown in Figs. 7a–c, the cascade-PI controller takes 30*ms* time for stabilizing the output voltage during start-up, SMC takes 40*ms* time to settle while producing peak overshoot of $58.33\%$, whereas the proposed ABSC achieves the same target in merely 16*ms*. Similarly, the Figs. 7a–c also indicate the inductor current response during startup under the cascade-PI, SMC and the proposed ABSC controller. It can be clearly seen that the inductor current under proposed control settles within 6*ms* when compared to the the cascade-PI control and SMC which settles in 22*ms* and 41*ms* respectively.

**Fig. 8:**
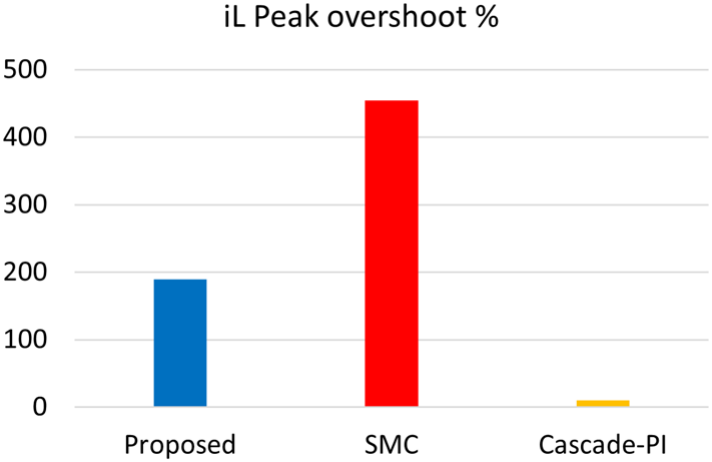
Bar graph representation of experimentation results.

**Fig. 9:**
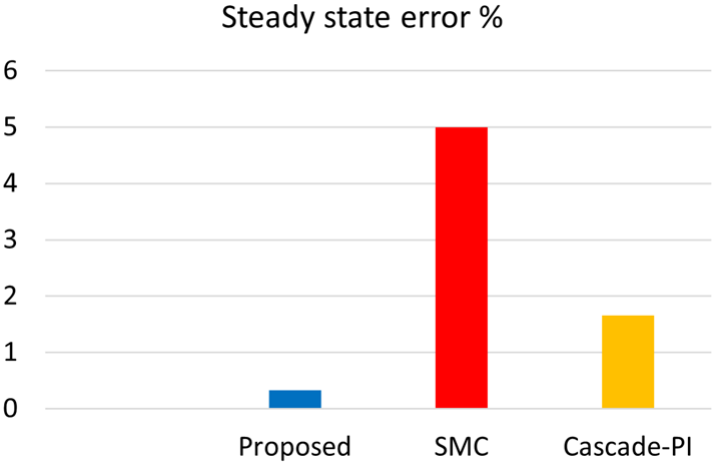
Bar graph representation of experimentation results.

Figure 8 shows the inductor peak overshoot plot for the proposed, SMC and Cascade-PI during start-up test condition. Figure 9 shows the steady state error plot for the proposed, SMC and Cascade-PI.

### Load change test

#### Load shift from $50-80W$

A 50 W resistive load operates during this test. An extra 30 W CPL is also present. The 50 W constant resistive load uses a $72 \Omega$ resistor. During this test scenario, the load shifts from 50 to 80 W, the proposed controller demonstrates a more compatible output voltage compared to other controllers like SMC and conventional cascade-PI. Standard cascade-PI control exhibits significant voltage undershoot. The output voltage dips by $41.6\%$. It requires 40 ms for stability. Whereas, the SMC takes 10 ms time to settle but produces the steady state error of intolerable 12 V. On the other hand, the proposed ABSC method results in an undershoot in output voltage, peaking at $20.8\%$, and stabilizing in 13 ms. In comparison, the proposed controller’s inductor current stabilizes within 12 ms, whereas the cascade-PI takes 15 ms for the same task.

**Fig. 10:**
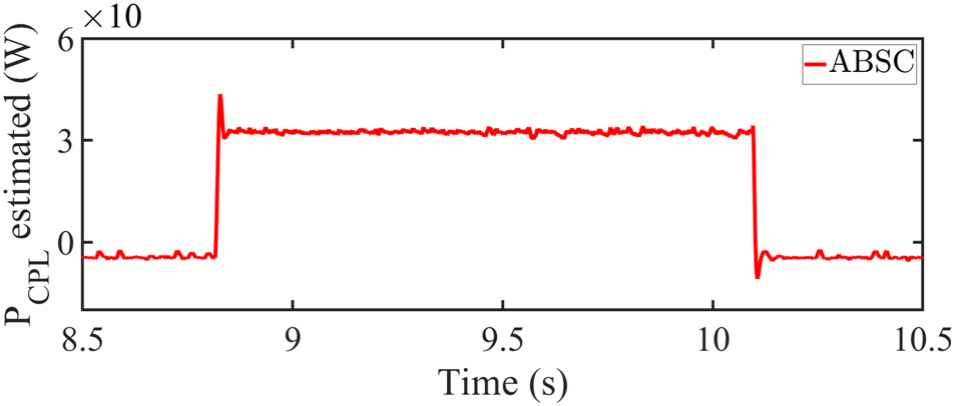
Estimated value of $P_{CPL}$ from 0 to 30 W using ABSC.

Conversely, a reduction in load from 80 to 50 W shows the proposed controller offers improved output voltage stability. The cascade-PI controller demonstrates less stability in this scenario. The cascade-PI shows a $25\%$ spike in output voltage and stabilizes in 38 ms. Whereas, the SMC takes 6*ms* time to settle. While the proposed ABSC stabilizes in 10 ms, while producing $15.63\%$ peak overshoot in the output voltage. The current in the inductor with the proposed controller stabilizes in merely 10*ms*. In comparison, the cascade-PI controller takes up to 20*ms* to achieve stability. On the other hand, the SMC takes 10*ms* time to settle to the steady-state value of current. The corresponding plots during this test scenario showing the output voltage and inductor current are presented in Figs. 7d–f. The load estimation plot of $P_{CPL}$ is shown in Fig. 10 during the load change test.

#### Load shift from $50-110W$

A 60W CPL was introduced. This addition supplemented a 50 W continuous resistive load. This setup is shown in Figs. 7g–i. The proposed controller under evaluation demonstrates a more consistent output voltage when the load transitions from 50 to 110 W. The output voltage peak overshoot, as indicated by ABSC, reaches $41.67\%$, and stabilizes after 24 ms. The inductor current stabilizes in 16 ms with our controller. When the load reduced from 110 to the 50 W, the proposed ABSC controller maintains the output voltage stable with only minor variations. Further, the proposed method reveals an output voltage with undershoot of $31.25\%$ while stabilizing after 14 ms. In contrast, the inductor current settles in a significantly lower 12 ms. The load current (indicated in green color) in Figs. 7g–i represents the current flowing through the load resistor. When examining load changes in Figs. 7d–f and g–i, it is clear that the ABSC causes minimal fluctuations in the resistive load current upon the introduction of $P_{CPL}$ into the system. However, these variations are more pronounced during transient conditions when utilizing the cascade-PI, as depicted in these figures. This clearly shows that, introducing $P_{CPL}$ into the system clearly causes fluctuations in the resistive load current, which the ABSC technique minimizes. Table 2 shows a numerical comparison. It covers the start-up and load change tests.

**Fig. 11:**
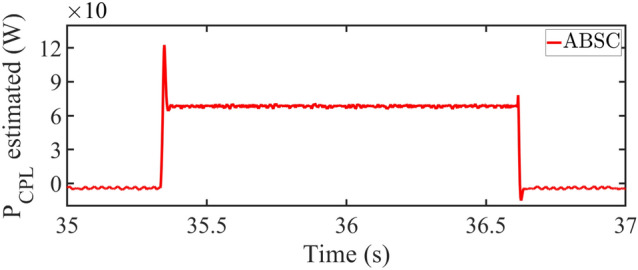
Estimated value of $P_{CPL}$ from 0 to 60 W using ABSC.

**Fig. 12:**
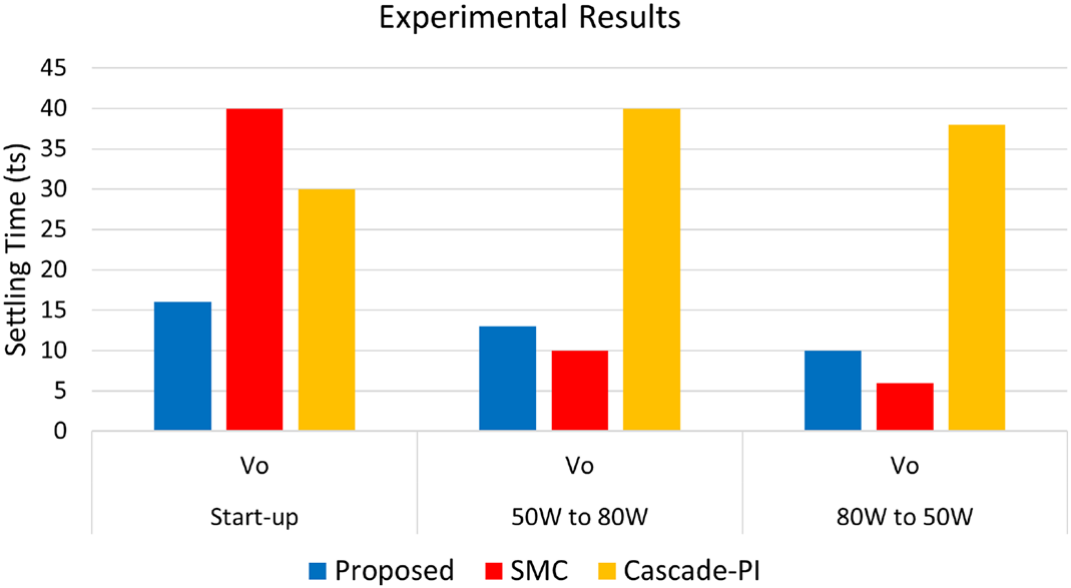
Represents the bar graph of the experimental results obtained during start up and load changes.

The load estimation utilizing ABSC, the analysis of $P_{CPL}$ during load variation is illustrated in Fig. 11. This plot visualizes the estimates for a $P_{CPL}=60W$ load condition. Similarly, the bar graph representation of the experimentation results under start-up and CPL change are presented in Figs. 8 and 12 depicts the bar graph of inductor current during start-up action. Finally, the steady-state errors during start-up are plotted in Fig. 9.

The cascade-PI control is ineffective in these conditions, causing the system to become unstable, as in Ref. 17. Instability arises when a constant resistive load is less than a CPL, any slight fluctuation in output voltage can lead to instability. This is evident in Fig. 2, where a negative incremental impedance occurs when $v_{o} < V_{o}$. Consequently, the necessary condition for stability is not fulfilled. In situations where $P_{CVL} < P_{CPL}$, even a small disturbance in output voltage can trigger system instability^17^.

#### Step by step varying CPL from 50 to 110*W*

**Fig. 13:**
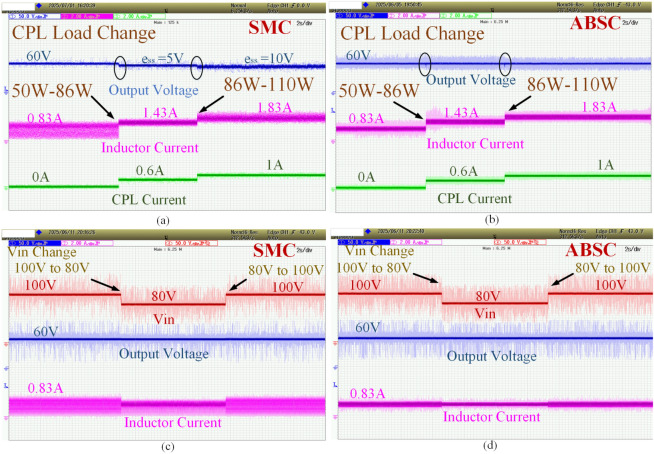
The varying CPL load response varying from the 50 to 86 W and 86 W to 110 W (**a**) SMC, (**b**) proposed ABSC; input voltage change response from 100 to 80 V and vice versa for both the (**c**) SMC (**d**) proposed ABSC.

In this test scenario the CPL is adding initial load of 50 W constant resistive load to 36 and 60W, making the over all load of 86 and 110W respectively. During this test, both the inductor current profile, CPL current profile are shown in Figs. 13a and b, which is varying from 0.83 to 1.43 and 1.83 A respectively. Comparison is made for SMC and the proposed ABSC methods. Here, by clean observation, the SMC control method is ineffective and is not maintaining the constant voltage when the addition of CPL. Whereas in the case of proposed ABSC method, it can withstand the variations in CPL and maintains the constant voltage. This can be clearly observed from Fig. 13b.

**Table 2:** Performance metrics during start-up and CPL change.

Controller	0 to 60V		50 to 80W		80 to 50W		50 to 110W		110 to 50W	
	$v_{o}$	$i_{L}$	$v_{o}$	$i_{L}$	$v_{o}$	$i_{L}$	$v_{o}$	$i_{L}$	$v_{o}$	$i_{L}$
	$t_{s}(ms)$	$t_{s}(ms)$	$t_{s}(ms)$	$t_{s}(ms)$	$t_{s}(ms)$	$t_{s}(ms)$	$t_{s}(ms)$	ts(ms)	$t_{s}(ms)$	$t_{s}(ms)$
Proposed	16	6	13	12	10	10	24	16	14	12
SMC	40	41	10	6	6	10	6	8	2	4
Cascade-PI	30	22	40	15	38	20	Unstable			

### Input voltage change test

In this test condition the input from the Programmable DC Power supply has changed from the voltage level of 100 to 80 V and vice versa. Here, the plots are shown for converters input voltage, output voltage and variation in the inductor current profile. Figure 13c and d shows the comparison of the both SMC and proposed ABSC method during this test condition. There is very minimal variation observed in the converters output voltage. By clean observation, the proposed ABSC method‘s inductor current profile is better than the SMC can be identified.

## Remark 2

he proposed ABSC method, while effective, also presents certain limitations. First, the tuning of controller gains and adaptation parameters is critical, as real-time disturbances can pose challenges when applied to converters with complex dynamics, despite the solid theoretical foundation provided by Lyapunov-based design. Second, the real-time estimation of CPL dynamics increases computational demand compared to classical PI controllers. Although our implementation on the dSPACE DS1104 platform successfully meets the requirements, the complex converters with higher switching frequencies may need a more powerful digital processors. Finally, this study’s validation is limited to a 120 W buck converter; scaling the approach to higher-power systems introduces additional concerns such as device non-idealities, thermal effects, and electromagnetic interference, all of which may impact controller performance.

## Conclusion

This article aims to present and assess an adaptive backstepping control method specifically designed to improve the stability of the DC-DC buck converter in a CPL system. The approach taken in this study involved a thorough series of experimental tests to evaluate how well the proposed controller works compared to a traditional cascade-PI controller and the SMC. The results from these tests demonstrate that the proposed controller performs better in handling the unexpected nonlinear load disturbances that challenge the converter performance during both the transient and steady-state conditions.

The effective use of this proposed control method in controlling the DC-DC buck converter, which powers both a resistive load and a CPL system, highlights its usefulness in various situations, especially in DC micro grids that operate in remote or difficult locations. This flexibility and efficiency demonstrate the practical importance of the proposed control method, making it a viable option for addressing stability and performance issues in DC-DC power converter configurations driving CPL.

### Future work

This proposed control method uses Adaptive Backstepping Control for a buck converter. It powers a CPL and resistive load. This versatile control method is not limited to the buck converter. It can be successfully applied to other types of power converters. This broad applicability enhances its practical value. Currently, the system’s focus is on estimating the CPLs power consumption. This estimation is a key component of the adaptive control loop. Future enhancements to this control system are planned. A significant improvement would involve estimating the input voltage. Accurate input voltage estimation is crucial. It directly impacts the overall performance of the power converter system.

## Data Availability

The datasets used and analyzed during the current study available from the corresponding author on reasonable request.
